# Phenolic Compounds from the Rhizomes of *Smilax china* L. and Their Anti-Inflammatory Activity

**DOI:** 10.3390/molecules22040515

**Published:** 2017-04-03

**Authors:** Cheng Zhong, Deng Hu, Lian-Bing Hou, Lu-Yao Song, Ying-Jun Zhang, Yang Xie, Li-Wen Tian

**Affiliations:** 1 School of Pharmaceutical Sciences, Southern Medical University, Guangzhou 510515, China; vanbenchat@126.com (C.Z.); hudeng19900922@126.com (D.H.); 2 Department of Pharmacy, Nanfang Hospital, Southern Medical University, Guangzhou 510515, China; lianbingh@hotmail.com (L.-B.H.); songluyao.nice@163.com (L.-Y.S.); 3 State Key Laboratory of Phytochemistry and Plant Resources in West China, Kunming Institute of Botany, Chinese Academy of Sciences, Kunming 650201, China; zhangyj@mail.kib.ac.cn

**Keywords:** *Smilax china*, phenolic compounds, anti-inflammatory activity

## Abstract

A new triflavanoid, kandelin B-5 (**1**), was isolated from the rhizomes of *Smilax china* L., together with six known phenylpropanoid substituted flavan-3-ols (**2**–**7**), nine flavonoids (**8**–**16**), two stilbenoids (**17**, **18**), and two other compounds (**19**, **20**). The structure of compound **1** was determined on the basis of 1D, 2D NMR and HR-ESI-MS data, as well as chemical method. Compounds **2**–**5**, **8**–**12**, **15**, **17**, and **19** were evaluated for anti-inflammatory activity. Only compounds **10**, **15** and **17** showed slightly IL-1β expression inhibitory activities on LPS induced THP-1 cells, with inhibition rate of 15.8%, 37.3%, and 35.8%, respectively, at concentration of 50 μg/mL.

## 1. Introduction

*Smilax china* L. (Liliaceae), a perennial climbing deciduous shrub, is widely distributed in Southern China, and Southeast Asian countries. The leaves of *S. china* are used as detoxication agent in folk China [1]; while the rhizomes of *S. china*, called “Jin Gang Teng”, are collected by Chinese Pharmacopoeia with the efficacy of carminative, diaphoretic, and circulative [2]. Previous studies on the title species have disclosed the presence of steroidal saponins [3], flavonoids [4,5], phenylpropanoids [6], and stilbenoids [7,8]. These isolated compounds showed a wide spectrum of activities, such as immunosuppressive activity [9], anti-oxidative activities [10], anti-inflammatory activities [3,11].

**Figure 1:**
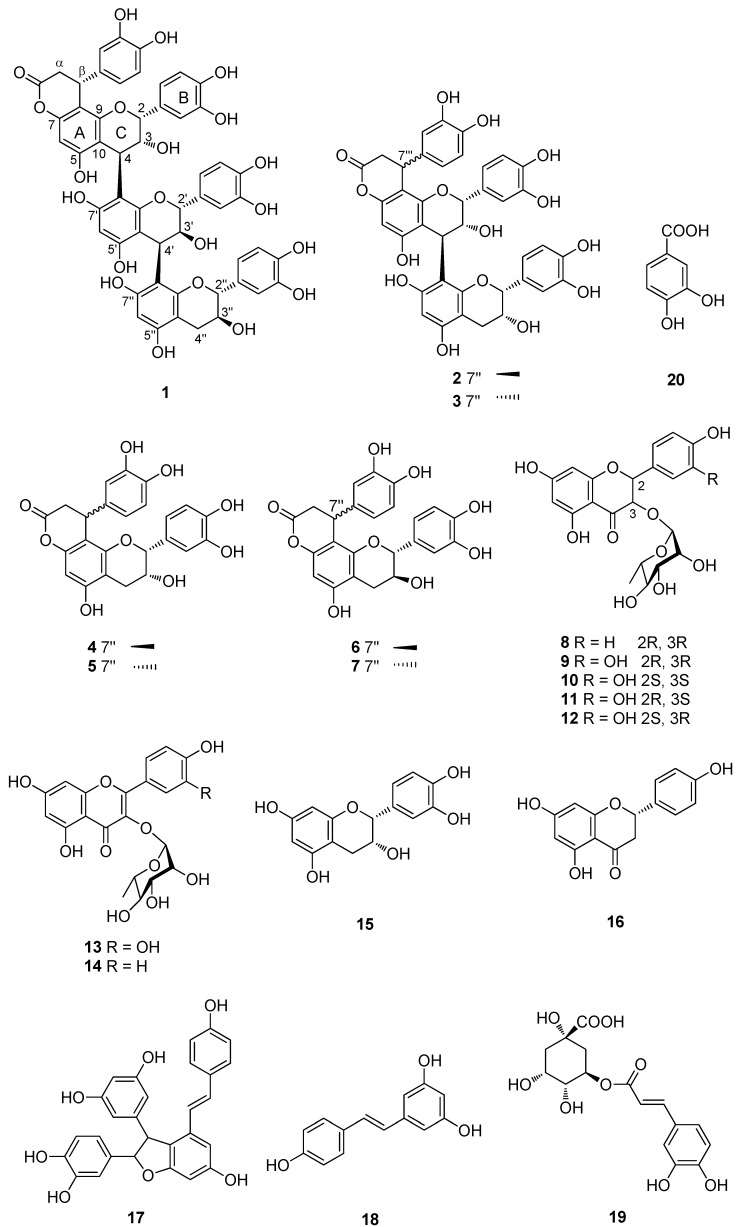
Chemical structures of **1**–**20**.

The pharmaceutical preparations of *S. china* are widely used in clinic for the treatment of chronic pelvic inflammatory disease, a kind of chronic inflammation in the female genital organs, connective tissues and pelvic peritoneum. Recently, we reported five known flavonoids from the anti-chronic pelvic inflammation fraction of *S. china* using high speed counter current chromatography [12,13]. Further detail chemical investigation on the title species led to the isolation of a new triflavanoid (**1**) and 19 known phenolic compounds (**2**−**20**) (Figure 1). Compounds **2**–**5**, **8**–**12**, **15**, **17**, and **19** were evaluated for their anti-inflammatory activity. Herein we describe the isolation and structure elucidation of these compounds as well as their anti-inflammatory activities.

## 2. Results and Discussion

The ethanol extract of the rhizomes of *S. china* was partitioned with Ethyl Acetate (EtOAc), and then subjected to column chromatography (CC) over MCI gel CHP 20P, Sephadex LH-20, Toyopearl HW 40F, silica gel, and preparative HPLC to yield a new triflavanoid (**1**), together with 19 known compounds. The known compounds were identified as cinchonain IIa (**2**) [14], cinchonain IIb (**3**) [14], cinchonain Ia (**4**) [15], cinchonain Ib (**5**) [15], catechin-[8,7-*e*]-4β-(3,4-dihydroxyphenyl)-dihyro-2(3*H*)-pyranone (**6**) [15], catechin-[8,7-*e*]-4α-(3,4-dihydroxyphenyl)-dihyro-2(3*H*)-pyranone (**7**) [15], engeletin (**8**) [16], astilbin (**9**) [17], neoastilbin (**10**) [17], isoastilbin (**11**) [17], isoneoastilbin (**12**) [17], quercetin-3-*O*-α-L-rhamnopyranoside (**13**) [12], luteolin-3-*O*-α-L-rhamnopyranoside (**14**) [18], (-)-epicatechin (**15**), 5,7,4′-trihydroxyflavonone (**16**), scirpusin A (**17**) [4], resveratrol (**18**) [4], chlorogenic acid (**19**) [19], and protocatechuic acid (**20**) [19], by comparison of their physicochemical data with those reported in the literature. Of them, compounds **2**–**3**, **14**, **16**, and **20** were isolated from the genus *Smilax* for the first time.

**Figure 2:**
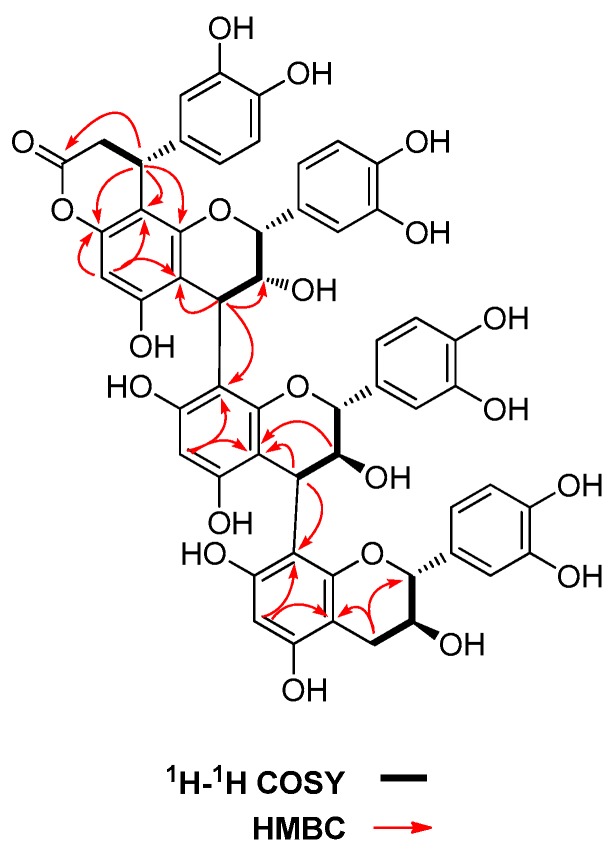
Key ^1^H-^1^H COSY and HMBC correlations of **1**.

Compound **1** was isolated as white amorphous powder. The molecular formula, C_54_H_44_O_21_, was determined on the basis of the HRESIMS data (*m*/*z* 1027.2285 [M − H]^−^; calcd. 1027.2297). The ^1^H-NMR data of **1** showed three singlets at δ_H_ 6.10, 5.90, 5.90, arising from the A-rings, three pairs of signals due to the flavan C_2_-H and C_3_-H at δ_H_ 5.17 (br.s, H-2), 4.48 (d, *J* = 7.4 Hz, H-2′), 3.83 (m, H-3′), 3.73 (d, *J* = 9.2 Hz, H-2′′), 3.71 (br.s, H-3), and 3.41 (m, H-3′′), two singlet methine signals at δ 4.51 (s, H-4′), 4.34 (s, H-4), and a methylene signal at δ_H_ 2.40 (br.d, *J* = 15.6 Hz, H-4′′α), 2.05 (dd, *J* = 7.0, 15.6 Hz, H-4′′β). The connectivities of these protons on C-rings were identified by ^1^H-^1^H COSY experiment (Figure 2). The above evidence indicated the presence of a triflavanoid moiety, which was consistent with ^13^C-NMR data. The ^13^C-NMR showed three aromatic methine carbons at δ_C_ 95.7, 95.5, 94.3, nine aliphatic carbons δ_C_ 82.1, 81.4, 75.6, 70.8, 68.2, 66.7, 36.3, 35.9, 30.7, and overlapped carbon signals arising from four 3,4-dihydroxybenzyl rings. The chemical shifts of the C-2 carbons, as well as the coupling patterns of the C_2_ proton signals suggested that the triflavanoid contains an epicatechin and two catechin moieties [20]. The ^13^C-NMR coupled with HSQC data showed the presence of an ester (δ_C_ 169.0), a methine (δ_C_ 33.6; δ_H_ 3.81, m), and a methylene [δ_C_ 37.0; δ_H_ 2.84 (dd, *J* = 6.0, 16.0 Hz), 2.25(dd, *J* = 10.0, 16.0 Hz)], suggesting the existence of a phenylpropanoid moiety. The phenylpropanoid moiety was connected with A-ring of the upper flavan-3-ol unit since all the protons on A rings showed singlets in the ^1^H-NMR spectrum [20]. Additionally, the phenylpropanoid moiety was presumed to be connected with C-8 position of upper flavan-3-ol unit as indicated by the carbon chemical shift of C-6 and C-8 [δ_C_: 96.0 (C-6), 105.2 (C-8) for C-8 substituted isomer; δ_C_: 106.2 (C-6), 99.4 (C-8) for C-6 substituted isomer] [21]. The two singlet methine signals at δ 4.51 (H-4′), 4.34 (H-4) indicated two β linkages of three flavan-3-ol units [14]. The 1D NMR of compound **1** was very similar to that of cinchonain IIa (**2**) or IIb (**3**), suggesting the C(4), C(8′)-linkages of flavan-3-ol moieties. The obviously up-field shifted of H-2′′ in the terminal catechin unit as comparing with catechin, attribute to the deshielding effect of phenyl group on the middle flavan-3-ol unit, suggested the C(4′), C(8′′)-linkages of middle and terminal flavan-3-ol units.

**Figure 3:**
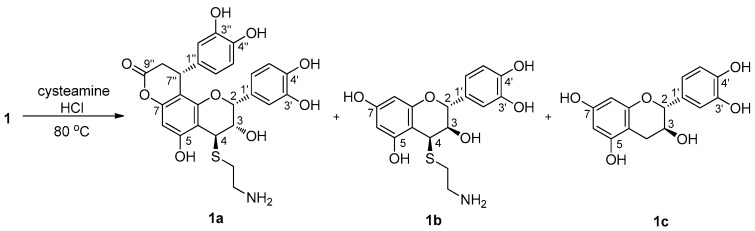
Acid-catalyzed thiolysis of **1**.

The constitution and points of the interflavanoid linkages in **1** were confirmed by hydrochloric acid-catalyzed degradation with cysteamine (Figure 3). Complete degradation of **1** afforded 4β-(2-aminoethylthio)-cinchonain Ib (**1a**), 4β-(2-aminoethylthio)-catechin (**1b**) [22], and catechin (**1c**), establishing that it consists of cinchonain Ib and catechin units. **1a** showed strong positive cotton effects at 230 nm and negative cotton effect at 250 nm, indicating that the phenyl group of phenylpropanoid unit was α-oriented. Thus, the structure of **1** was elucidated as showed in Figure 1.

Interleukin-1β (IL-1β), an important mediator of the inflammatory response, is involved in a variety of cellular activities, including cell proliferation, differentiation, and apoptosis. Compounds **2**–**5**, **8**–**12**, **15**, **17**, and **19** were evaluated for their IL-1β expression inhibitory activities on lipopolysaccharide (LPS) induced THP-1 cells. Compounds **10**, **15**, and **17** showed slightly inhibitory activities, with inhibition rate of 15.8%, 37.3%, and 35.8%, respectively, at concentration of 50 μg/mL. The other compounds showed no obvious activity at the same concentration. CCK-8 results revealed that these tested compounds showed no obvious cytotoxicities towards THP-1 cells at same concentration, indicating that the anti-inflammatory activities of compounds **10**, **15**, and **17** were not resulted from cytotoxic effects. Previous studies revealed the unique immunosuppressive activity of astilbin (**9**) [23]; While our results suggested that astilbin (**9**) and its isomers (**10**–**12**) didn’t directly inhibit the production of proinflammatory cytokines IL-1β. (-)-Epicatechin and stilbene are reported to show anti-inflammatory activities [24]. Consistently, our results showed that (-)-Epicatechin (**15**) and stilbene dimer, scirpusin A (**17**), inhibited IL-1β expression on the LPS induced THP-1 cells.

## 3. Materials and Methods

### 3.1. General Experimental Procedures

Optical rotation was measured with an MCP-500 polarimeter (Anton Paar). The UV spectrum was recorded with a TU-1810DSPC UV-Vis spectrometer (Puxi Tongyong). IR spectrum was measured on a IR Affinity-1 spectrophotometer (Shimadzu); CD was measuredon a Chirascan^TM^ (Applied Photophysics Ltd., Leatherhead, UK). NMR was acquired on an AV-400 spectrometer (Bruker) with TMS as internal standard, *J* in Hz; HRESIMS spectra were measured on the Orbitrap Fusion high resolution mass spectrometer (Thermo) equipped with ESI source. Preparative or semi-preparative HPLC were performed on a HPLC system equipped with a Waters 1525 pump and a Waters 2487 Dual Wavelength Detector using a Thermo hypersil C_18_ column (5 μm, 150 × 25.0 mm, i.d.) or Thermo hypersil C_18_ column (5 μm, 250 × 10 mm, i.d.). Open column chromatography was performed using silica gel (200–300 mesh, Qingdao Marine Chemical, Qingdao, China), Sephadex LH-20 (25–100 μm, Fiji), MCI gel CHP 20P (75–150 μm, Fiji), Toyopearl HW 40F (TOSOH, Tokyo, Japan) or ODS (40–63 μm, Merck, Kenilworth, NJ, USA).

### 3.2. Plant Materials

The rhizomes of *S. china* were purchased from Shenzhen Hongen Pharmaceutical Company, and were identified by one of the authors (L.B. Hou). The voucher specimen of this material (NO. SMU-NPC-201301) was deposited in Natural Product Chemistry Lab, School of Pharmaceutical Sciences, Southern Medical University, Guangzhou, China.

### 3.3. Extraction and Isolation

The rhizomes of *S. china* (6.0 kg) were refluxed with 95% ethanol at 75 °C for three hours, three times and filtered. The combined filtrate was concentrated to yield crude extracts (1.2 kg). The crude extracts were suspended in H_2_O, and then partitioned with EtOAc and n-BuOH consecutively. A portion (8.5 g) of the EtOAc-solution fraction (150 g) was subjected to MCI gel CHP 20P (MeOH/H_2_O, 1:9–10:0) CC to give seven fractions A-G. Fraction A (0.21 g) was chromatographed over MCI gel CHP 20P (MeOH/H_2_O, 1:9–4:6), and ODS (MeOH/H_2_O, 2:8–5:5) to afford **20** (4.0 mg). Fraction B (0.25 g) was chromatographed over Toyopearl HW 40F (MeOH/H_2_O, 3:7–8:2) to yield four sub-fractions, B1–B4. Sub-fraction B1 was subjected to MCI gel CHP 20P (MeOH/H_2_O, 2:8–6:4) to yield **19** (4.0 mg). Sub-fraction B2 was chromatographed over ODS (MeOH/H_2_O, 2:8–6:4), Toyopearl HW 40F (MeOH/H_2_O, 3:7–7:3), and Sephadex LH-20 (100% MeOH) to yield **15** (9.0 mg). Sub-fraction B4 was purified by preparative HPLC (gradient model, MeOH/H_2_O, 20–80% over 30 min, MeOH and H_2_O contained 0.1% TFA) to yield **1** (16 mg). Fraction C (4.08 g) was chromatographed over MCI gel CHP 20P (MeOH/H_2_O, 3:7–9:1) to yield three sub-fractions, C1-C3. Sub-fraction C2 was purified by Toyopearl HW 40F (MeOH/H_2_O, 4:6–1:0) CC, and preparative HPLC (gradient model, MeOH/H_2_O, 20–80% over 30 min, MeOH and H_2_O contained 0.1% TFA) to afford **2** (21 mg), and **3** (26 mg). Sub-fraction C3 was subjected to CC over Toyopearl HW 40F (MeOH/H_2_O, 4:6–1:0) to yield three sub-fractions, C3-1, C3-2, C3-3. Sub-fraction C3-2 was purified by semi-preparative HPLC (35% MeOH, isocratic model) to afford **9** (8 mg), **10** (40 mg), **11** (6 mg), and **12** (5 mg). Sub-fractions C3-3 was purified by semi-preparative HPLC (gradient model, MeOH/H_2_O, 20–80% over 30 min, MeOH and H_2_O contained 0.1% TFA) to yield **4** (21 mg), **5** (15 mg), **6** (4 mg), and **7** (6 mg). Fraction D (1.0 g) was chromatographed over MCI gel CHP 20P (MeOH/H_2_O, 4:6–1:0), Toyopearl HW 40F (MeOH/H_2_O, 4:6–1:0), and Sephadex LH-20 (100% MeOH) to afford **13** (42 mg). Fraction E (1.0 g) was chromatographed over MCI gel CHP 20P (MeOH/H_2_O, 4:6–8:2), Toyopearl HW 40F (MeOH/H_2_O, 4:6–8:2) to yield **8** (30 mg), and **16** (9.0 mg). Fraction F (0.4 g) was subjected to CC over MCI gel CHP20P (MeOH/H_2_O, 6:4–1:0), Sephadex LH-20 (100% MeOH), and silica gel (DCM/MeOH, 9:1–8:2) to afford **14** (12 mg), **17** (5 mg), and **18** (9 mg).

Kandelin B-5 (**1**): Amorphous powder, ${\left[\alpha \right]}_{D}^{25}$ = 86.5 (*c* = 0.18, MeOH); UV (MeOH), λ_max_ (logε): 227 (4.55), 282 (4.03) nm; IR(KBr)ν_max_ 3350, 1743, 1616, 1521, 1448, 1284, 1109 cm^−1^; ^1^H-NMR (400 MHz, DMSO-*d*_6_) and ^13^C-NMR (100 MHz, DMSO-*d*_6_): see Table 1; (-)-HRESIMS: 1027.2285 [M − H]^−^ (Calcd for C_54_H_43_O_21_, 1027.2297).

### 3.4. Thiolysis of Compound ***1***

A mixture of compound **1** (8 mg), cysteamine (20 mg), and 1 M aqueous hydrochloric acid (3 mL) was refluxed for 2 h with stirring. After removal of the solvent by evaporation under reduced pressure, the oily residue was applied to a Sephadex LH-20 column (DCM/MeOH, 1:1), and was further purified by semi-preparative HPLC (gradient model, MeOH/H_2_O, 10–90% over 30 min) to afford: **1a** (0.9 mg), **1b** (0.6 mg), and **1c** (0.4 mg).

4β-(2-aminoethylthio)-cinchonain Ib (**1a**): Amorphous powder, ${\left[\alpha \right]}_{D}^{25}$ = 41.8 (*c* = 0.09, MeOH); ^1^H-NMR (400 MHz, Acetone-*d*_6_): δ 6.91 (1H, d, *J* = 1.9 Hz, H-2′), 6.74 (2H, s, H-5′, 6′), 6.71 (2H, m, H-2′′, 5′′), 6.53 (1H, dd, *J* = 2.0, 8.1 Hz, H-6′′), 6.23 (1H, s, H-6), 5.27 (1H, br.s, H-2), 4.45 (1H, m, H-7′′), 4.17 (1H, br.s, H-4), 4.12 (1H, br.s, H-3), 3.07 (1H, dd, *J* = 6.9, 15.9 Hz, H-8′′a), 2.90 (1H, br.d, *J* = 15.9 Hz, H-8′′b), 3.80-2.80 (4H, m, S-CH_2_-CH_2_-N); (-)-ESIMS: *m*/*z* 526 [M − H]^−^.

4β-(2-aminoethylthio)-catechin (**1b**): Amorphous powder, ${\left[\alpha \right]}_{D}^{25}$ = 33.2 (*c* = 0.06, MeOH); ^1^H-NMR (400 MHz, Acetone-*d*_6_): δ 6.91 (1H, d, *J* = 1.9 Hz, H-2′), 6.75 (1H, d, *J* = 8.1 Hz, H-5′), 6.70 (1H, dd, *J* = 1.9, 8.1 Hz, H-6′), 5.91 (2H, s, H-6, 8), 5.31 (1H, d, *J* = 9.3 Hz, H-2), 4.00 (1H, m, H-3), 3.88 (1H, d, *J* = 6.6 Hz, H-4), 3.60–2.40 (4H, m, S-CH_2_-CH_2_-N).

catechin (**1c**): Amorphous powder; ^1^H-NMR (400 MHz, Acetone-*d*_6_): δ 6.91 (1H, d, *J* = 1.9 Hz, H-2′), 6.81 (1H, d, *J* = 8.1 Hz, H-5′), 6.77 (1H, dd, *J* = 1.9, 8.1 Hz, H-6′), 6.04 (1H, d, *J* = 1.9 Hz, H-8), 5.89 (1H, d, *J* = 1.9 Hz, H-6), 4.57 (1H, d, *J* = 7.8 Hz, H-2), 4.00 (1H, m, H-3), 2.93 (1H, dd, *J* = 5.5, 16.0 Hz, H-4a), 2.90 (1H, dd, *J* = 8.4, 16.0 Hz, H-4b).

### 3.5. Quantitation of Cytokine IL-1β

The human acute monocytic leukemia (THP-1) cells (Shanghai Cell Bank, C.A.S., Shanghai, China) were cultured in Dulbecco’s Modefied Eagles Medium (DMEM) containing 10% Fetal Bovine Serum (FBS) at 37 °C in a humidified atmosphere with 5% CO_2_. The cells were seeded into 96-well plates with density of 1 × 10^5^ cells per milliliter. Thereafter, cells were treated with sample solutions diluted with cell sustainable medium. After incubation for 1 h, the cells were treated with 1 μg/mL of LPS for another 24 h. The cells were treated with LPS alone or LPS plus dexamethasone as blank control and positive control, respectively. The supernatant was collected by centrifugation (14,000 rpm, 4 °C). The quantity of IL-1β in culture supernatant was determined by using Enzyme Linked Immunosorbent Assay (ELISA) kit specific for IL-1β [25]. The inhibition rates were calculated according to the formula:

Inhibition rate = [(C − T)/C] × 100%
(1)

where C is the average quantity of IL-1β of the blank control and T is the average quantity of IL-1β of the group with the test compound.

### 3.6. Cytotoxicity Assay

The effect of compounds **2**–**5**, **8**–**12**, **15**, **17**, and **19**, on the viability of THP-1 cells were evaluated using the Cell Counting Kit-8 (CCK-8) according to the manufacturer’s instructions. Briefly, THP-1 cells were seeded into 96-well plates at a density of 2.5 × 10^4^ cells/well in the presence of sample solutions (50, 100, 200 μM, respectively) or absence of compounds for 48 h. Thereafter, 10 μL CCK-8 solutions were added to each well for further incubation at 37 °C in 4 h. The optical density was measured at a wavelength of 450 nm using a microplate reader [26]. Cell viability was expressed as a percentage of the control.

## 4. Conclusions

In this study, a new triflavanoid, kandelin B-5 (**1**), was isolated from the rhizomes of *Smilax china* L., together with six known phenylpropanoid substituted flavan-3-ols (**2**–**7**), nine flavonoids (**8**–**16**), two stilbenoids (**17**,**18**), and two other compounds (**19**,**20**). The structure of compound **1** was determined on the basis of 1D, 2D NMR and HR-ESI-MS data, as well as chemical method. Compounds **2**–**5**, **8**–**12**, **15**, **17**, and **19** were evaluated for anti-inflammatory activities. Only neoastilbin (**10**), (-)-epicatechin (**15**), and scirpusin A (**17**) showed slight IL-1β expression inhibitory activities on LPS induced THP-1 cells.

## Figures and Tables

**Table 1:** ^1^H- (400 MHz) and ^13^C-NMR (100 MHz) data of **1** (in DMSO-*d*_6_, *J* in Hz, δ in ppm).

Position	δ_H_	δ_C_
2	5.17 br.s	75.6
3	3.71 br.s	70.8
4	4.34 br.s	36.3
6	5.90 s	94.0
8		108.0
10		99.5
2′	4.48 d (7.4)	81.4
3′	3.38 m	66.7
4′	4.51 br.s	35.9
6′	5.90 s	95.1
8′		108.0
10′		103.6
2′′	3.73 d (9.2)	82.1
3′′	3.41 m	68.2
4′′	2.84 dd (6.0, 16.0)	30.7
6′′	2.25 dd (10.0, 16.0)	95.7
8′′		107.7
10′′		100.4
α	2.40 br.d (15.6)	38.1
β	2.05 dd (7.0, 15.6)	33.9
-COO-		169.0

## Supplementary Materials

Supplementary materials are available online. The NMR spectra of **1**; the ^1^H-NMR, ESIMS, and CD spectra of hydrolysis products of **1**.
