# Supplementary material for: Phenolic Compounds from the Rhizomes of Smilax china L. and Their Anti-Inflammatory Activity
Source: Molecules. 2017 Apr 3;22(4):515. doi: 10.3390/molecules22040515 (PMC6154459; doi:10.3390/molecules22040515)
Supplement: Supplementary file 1 [file molecules-22-00515-s001.pdf]

# Supplementary Materials: Phenolic Compounds from the Rhizomes of *Smilax china* L. and Their Anti-Inflammatory Activity

**Cheng Zhong, Deng Hu, Lian-Bing Hou, Lu-Yao Song, Ying-Jun Zhang, Yang Xie and Li-Wen Tian**

## Contents of Supplementary Material


| No. | Contents: | Pages: |
|---|---|---|
| **Figure S1.** | $^{13}C$ NMR spectrum (100 MHz, DMSO-*d*$_6$) of compound **1**. | 3 |
| **Figure S2.** | $^{1}H$ NMR spectrum (400 MHz, DMSO-*d*$_6$) of compound **1**. | 4 |
| **Figure S3.** | HSQC spectrum of compound **1**. | 5 |
| **Figure S4.** | $^{1}H$-$^{1}H$ COSY spectrum of compound **1** | 6 |
| **Figure S5.** | HMBC spectrum of compound **1**. | 7 |
| **Figure S6.** | $^{1}H$ NMR spectrum (400 MHz, Acetone-*d*$_6$) of compound **1a** | 8 |
| **Figure S7.** | ESIMS spectrum of compound **1a** | 9 |
| **Figure S8.** | CD spectrum (MeOH) of compound **1a**. | 10 |
| **Figure S9.** | $^{1}H$ NMR spectrum (400 MHz, Acetone-*d*$_6$) of compound **1b**. | 11 |
| **Figure S10.** | $^{1}H$ NMR spectrum (400 MHz, Acetone-*d*$_6$) of compound **1c**. | 12 |

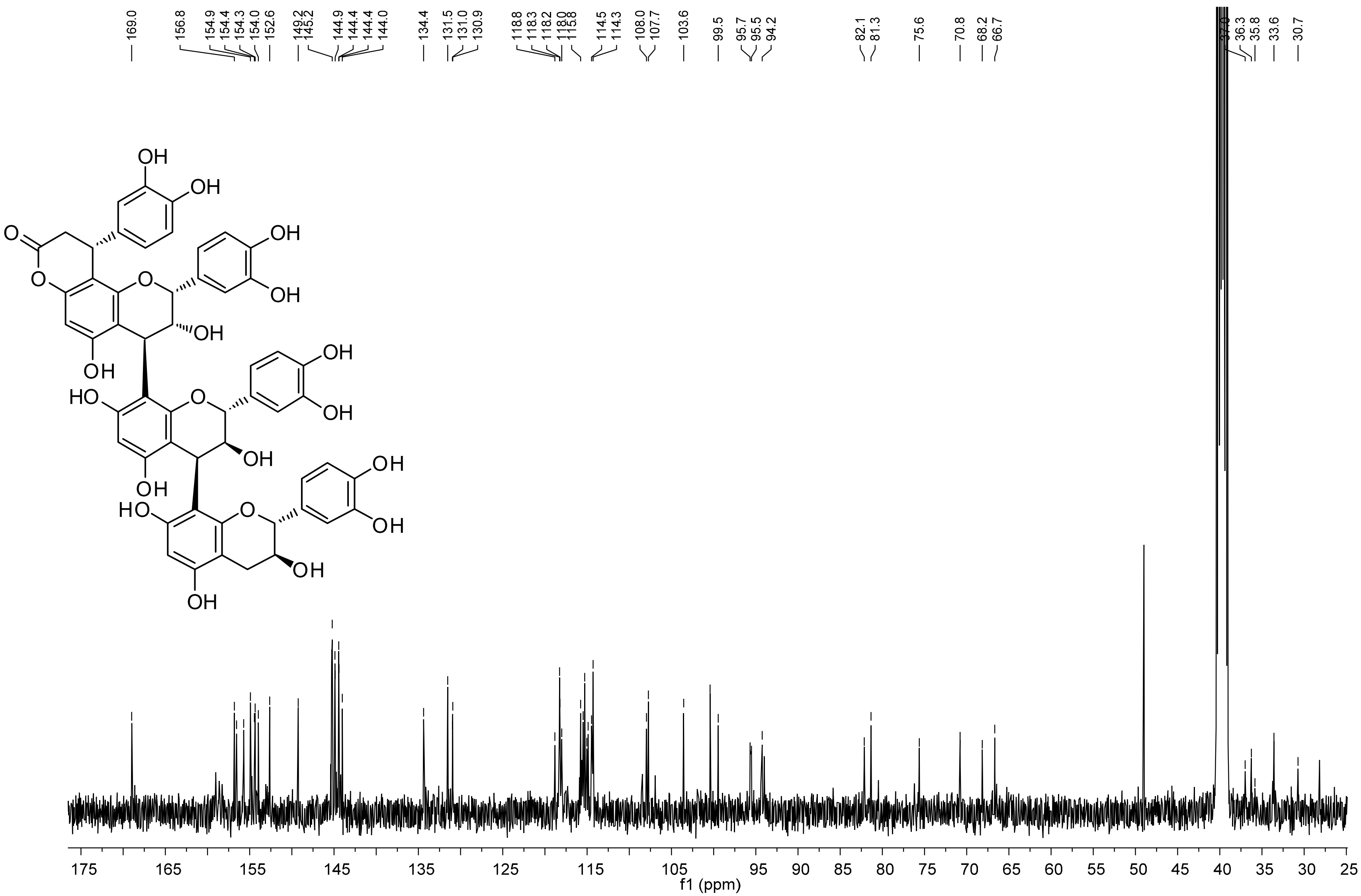


**Figure S1.** $^{13}$C NMR spectrum (100 MHz, DMSO-$d_6$) of compound **1**.

**Figure S2.** $^{1}$H NMR spectrum (400 MHz, $CDCl_3$) of compound **1**.

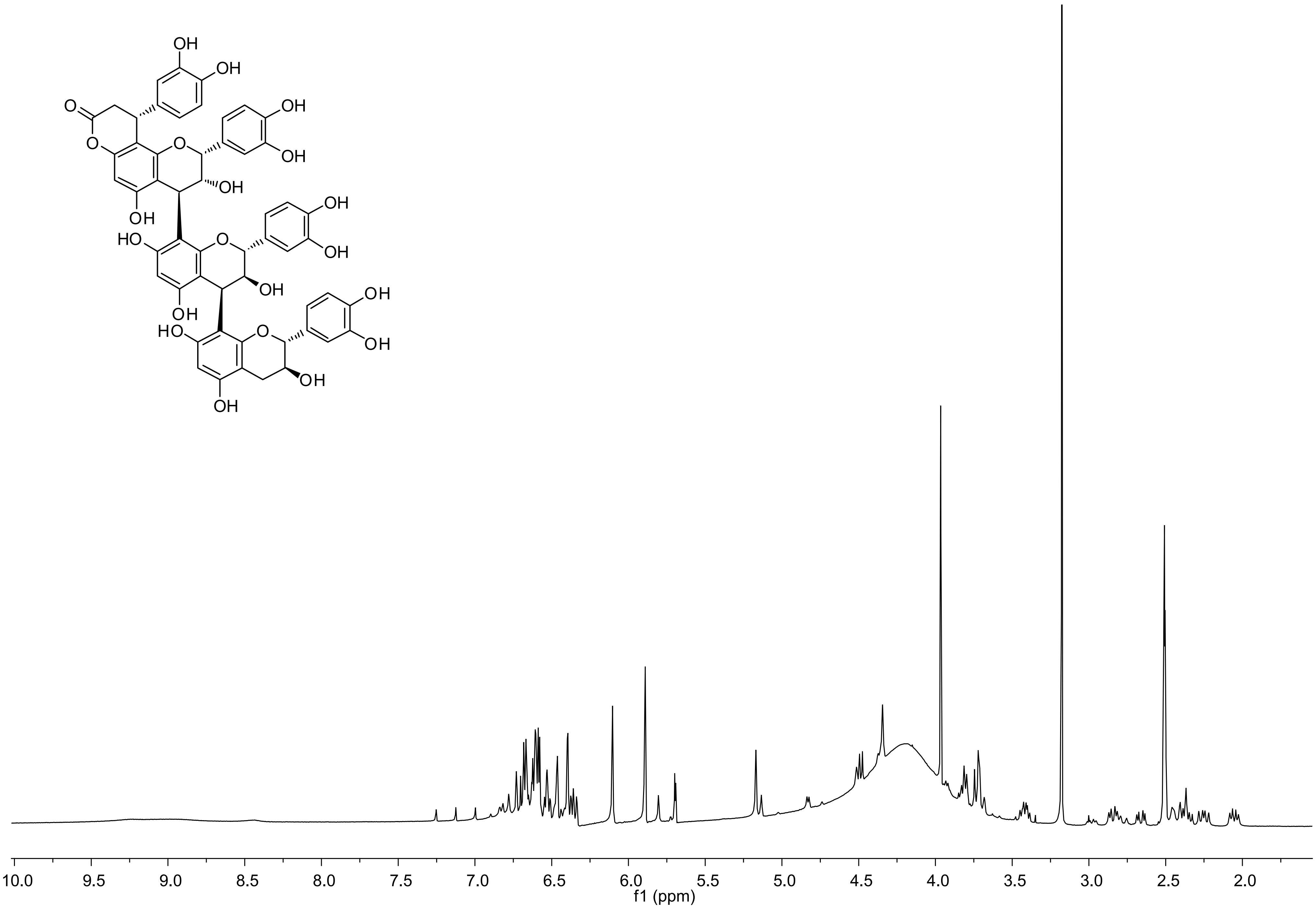
10.0 9.5 9.0 8.5 8.0 7.5 7.0 6.5 6.0 5.5 5.0 4.5 4.0 3.5 3.0 2.5 2.0
f1 (ppm)

**Figure S3.** HSQC spectrum of compound **1**.

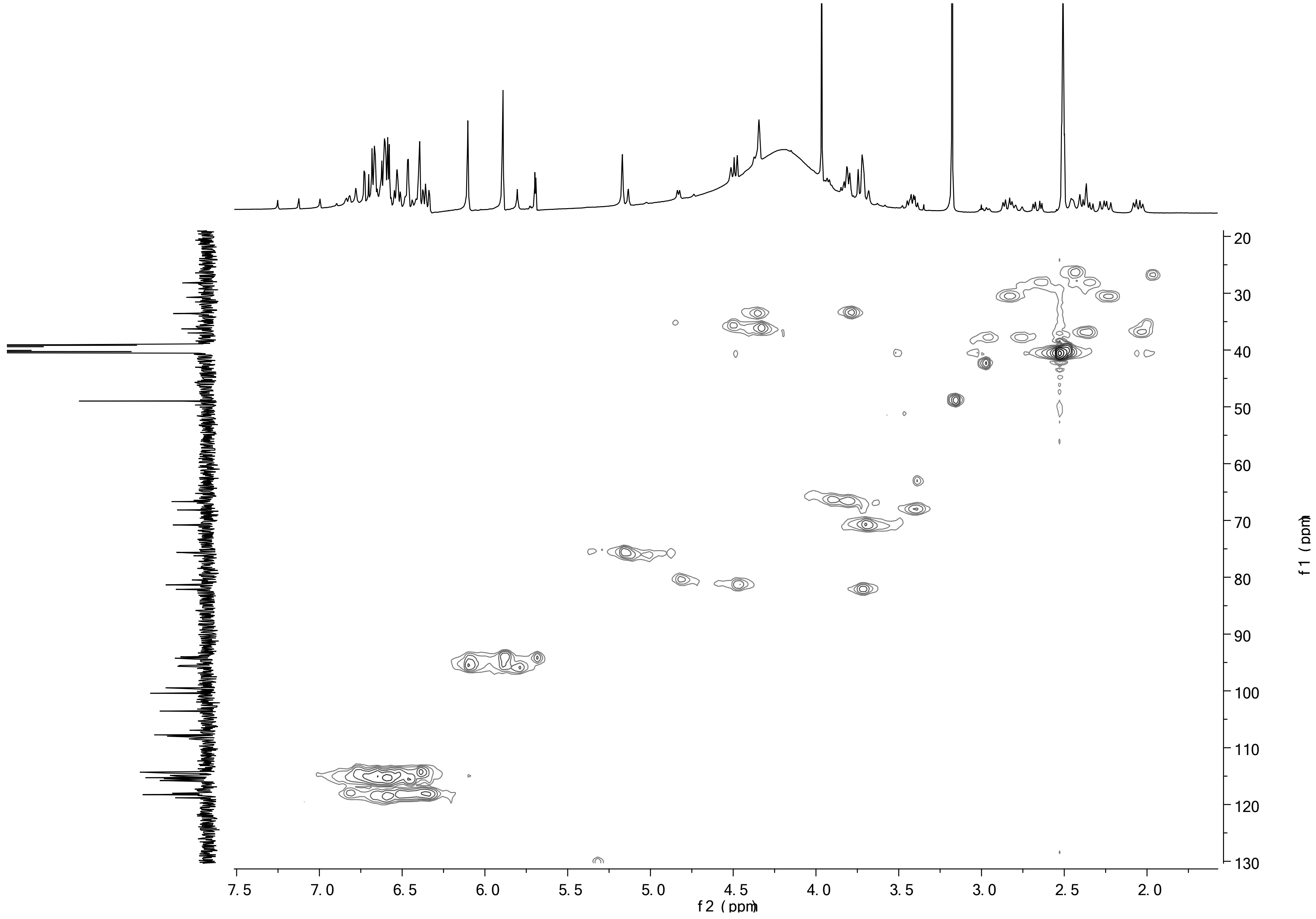


7

**Figure S4.** $^1H$-$^1H$ COSY spectrum of compound **1**

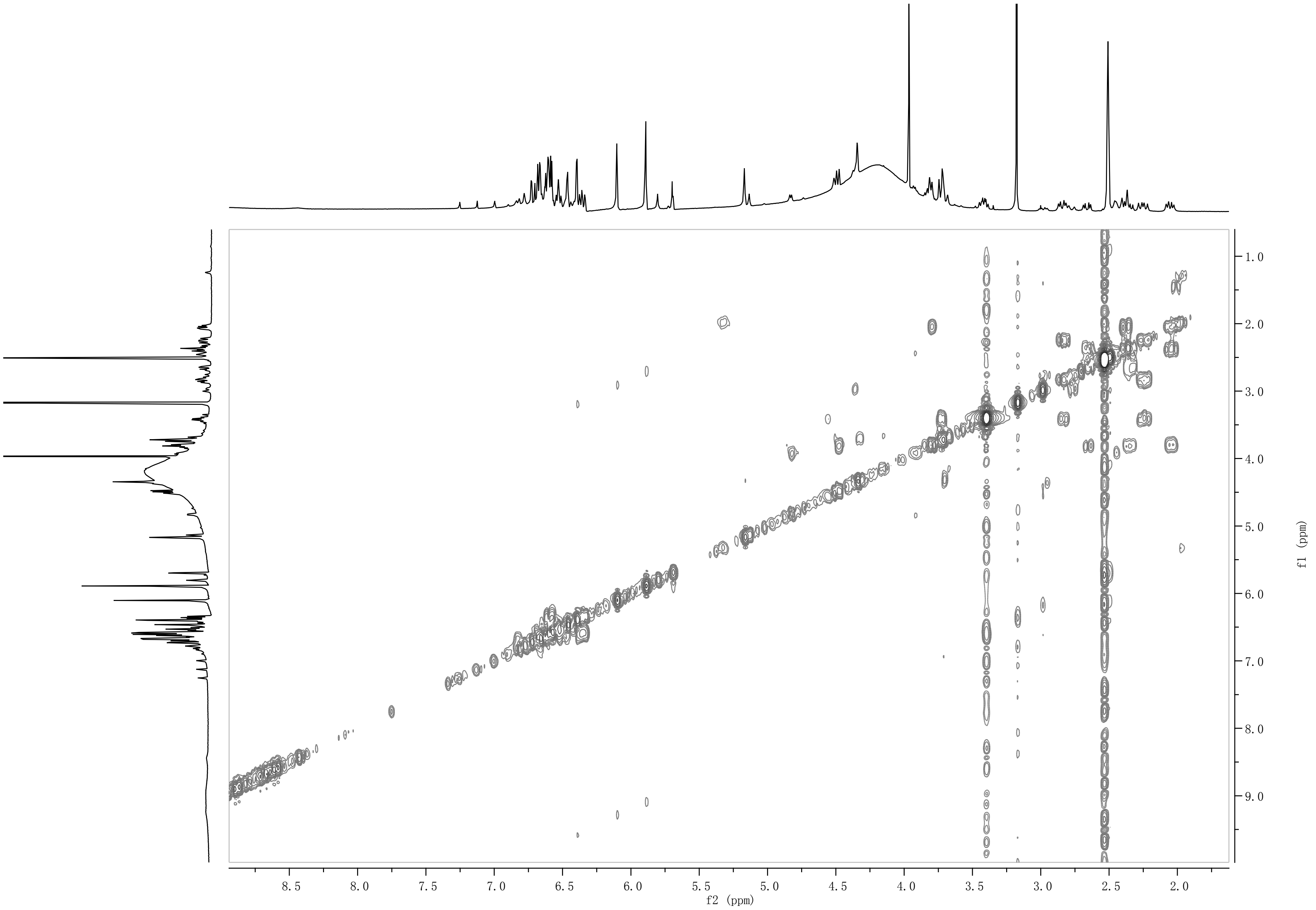
f2 (ppm)
f1 (ppm)

**Figure S5.** HMBC spectrum of compound **1**.

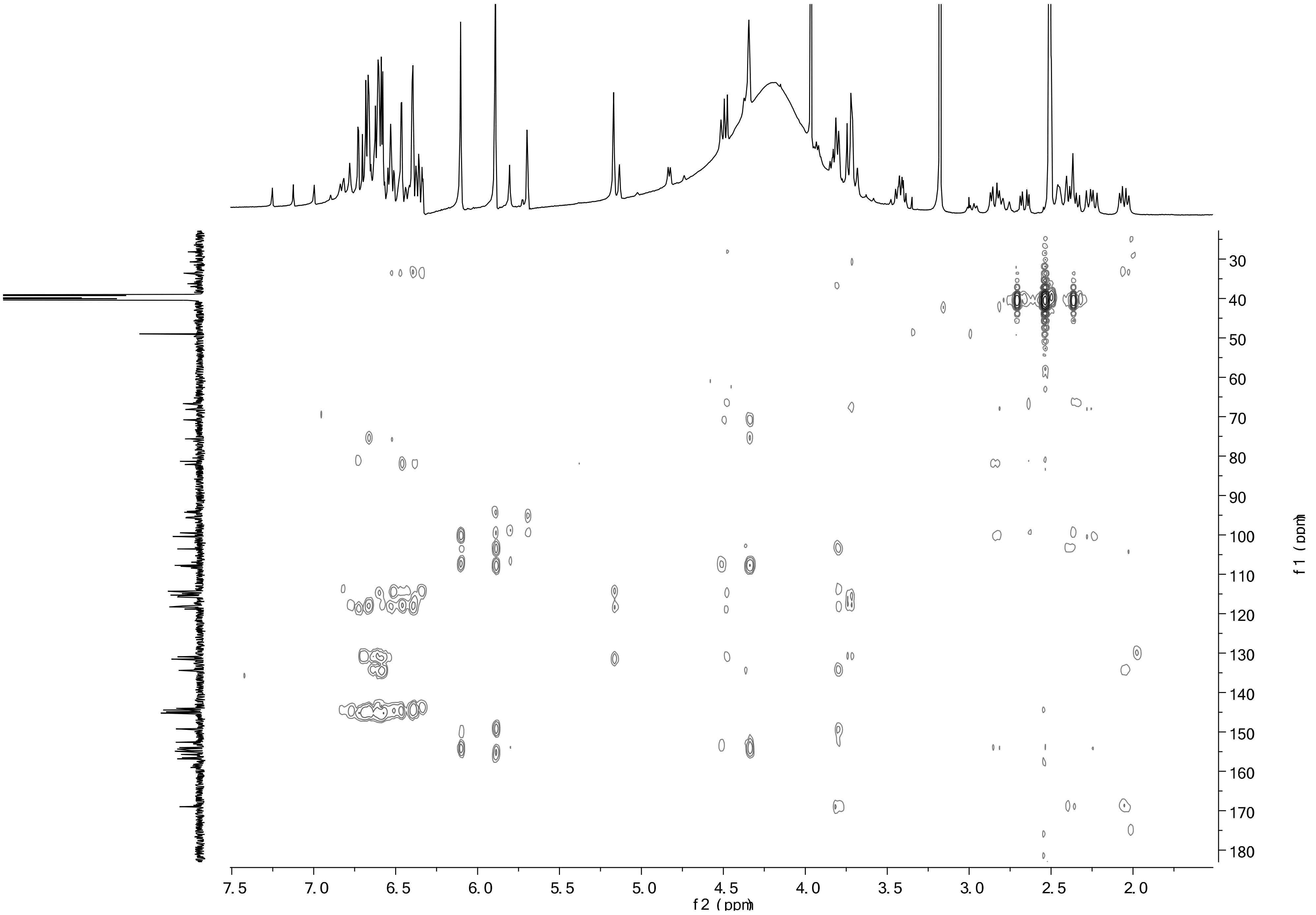
f1 (ppm)
f2 (ppm)

**Figure S6.** $^1$H NMR spectrum (400 MHz, acetone-$d_6$) of compound **1a**

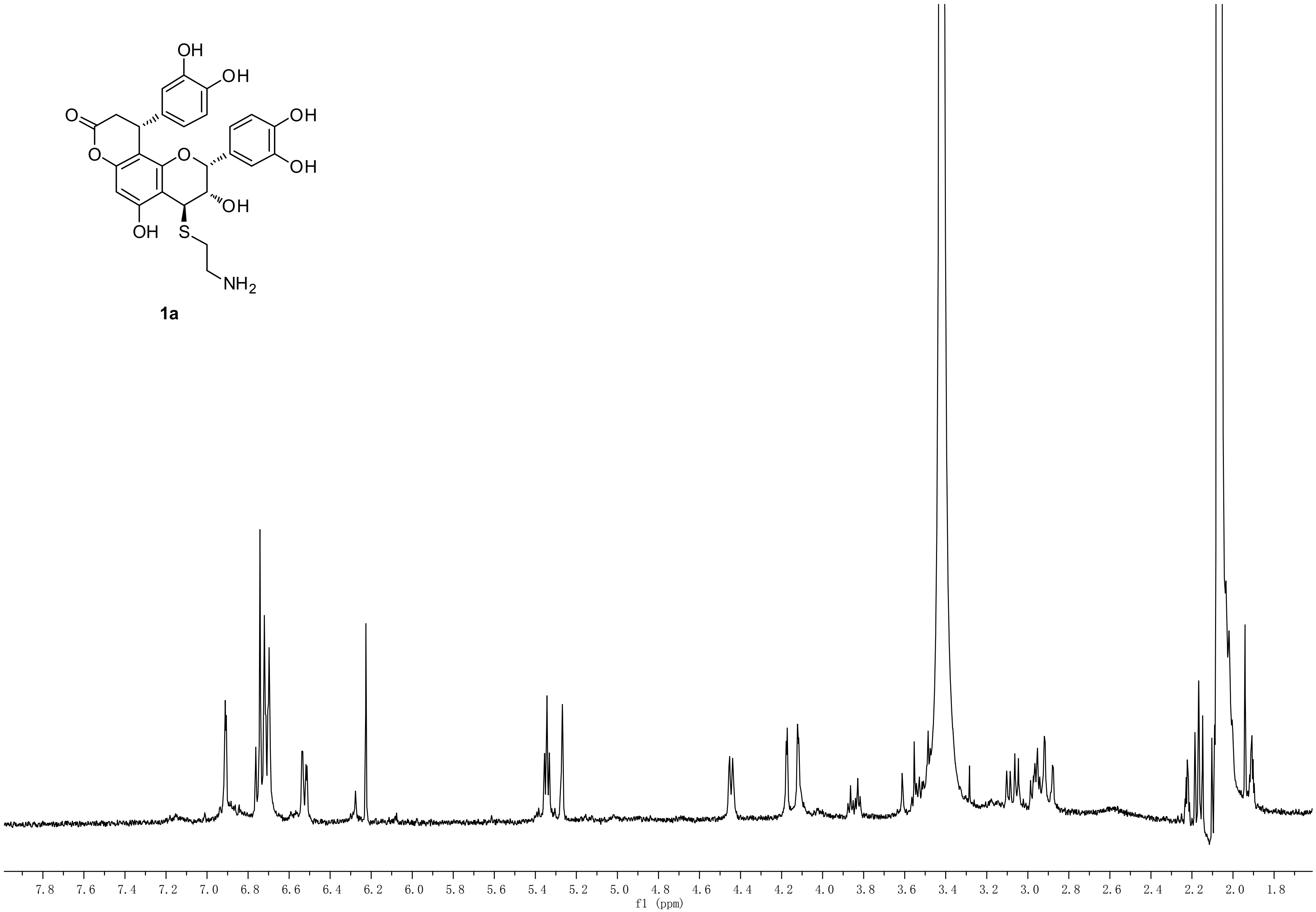

**Figure S7.** ESI-MS spectrum of compound **1a**

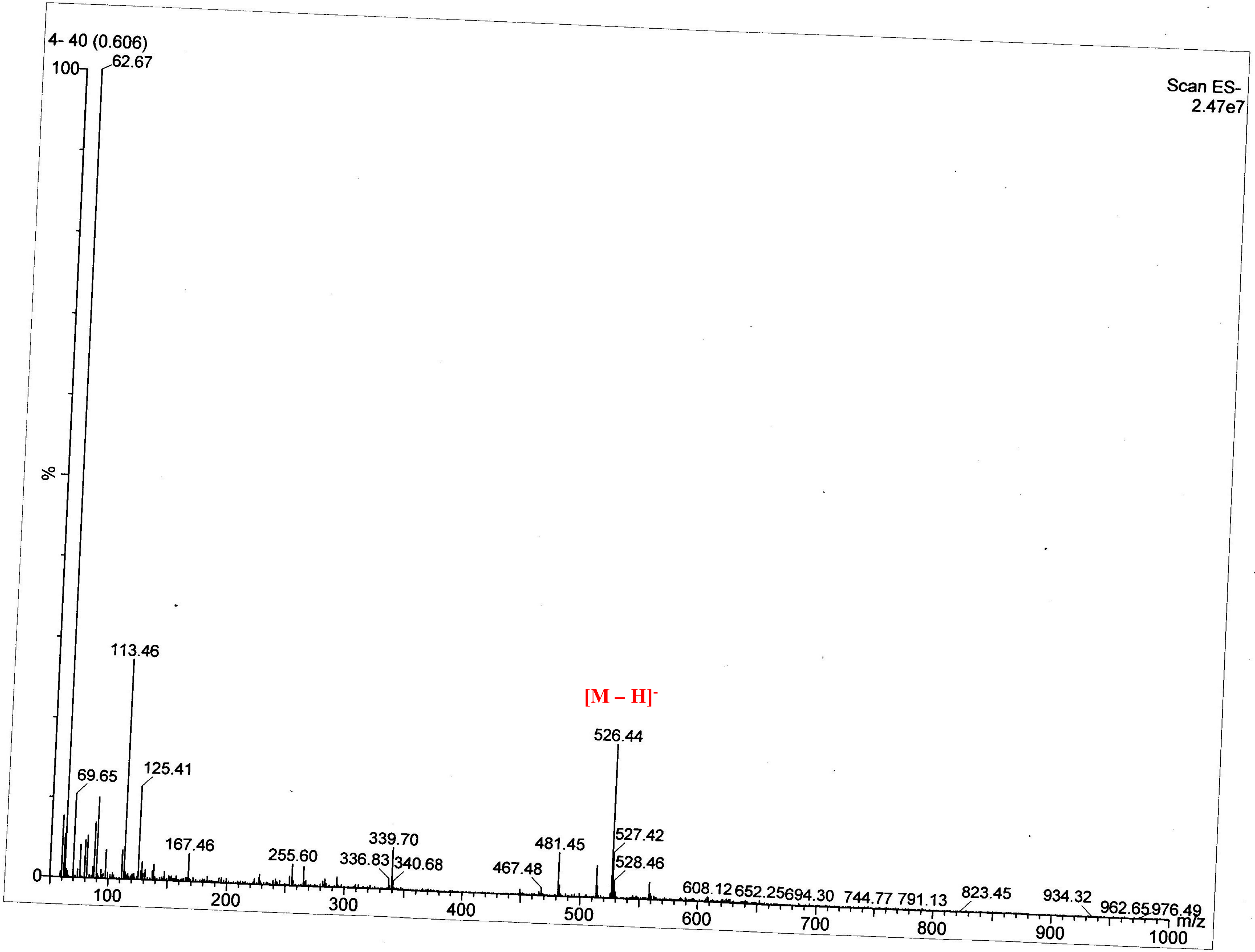

4- 40 (0.606)
Scan ES-
2.47e7
100
%
0
62.67
113.46
69.65
125.41
167.46
255.60
339.70
336.83
340.68
467.48
481.45
[M – H]-
526.44
527.42
528.46
608.12
652.25
694.30
744.77
791.13
823.45
934.32
962.65
976.49
m/z
100
200
300
400
500
600
700
800
900
1000

**Figure S8.** CD spectrum (MeOH) of compound **1a**.

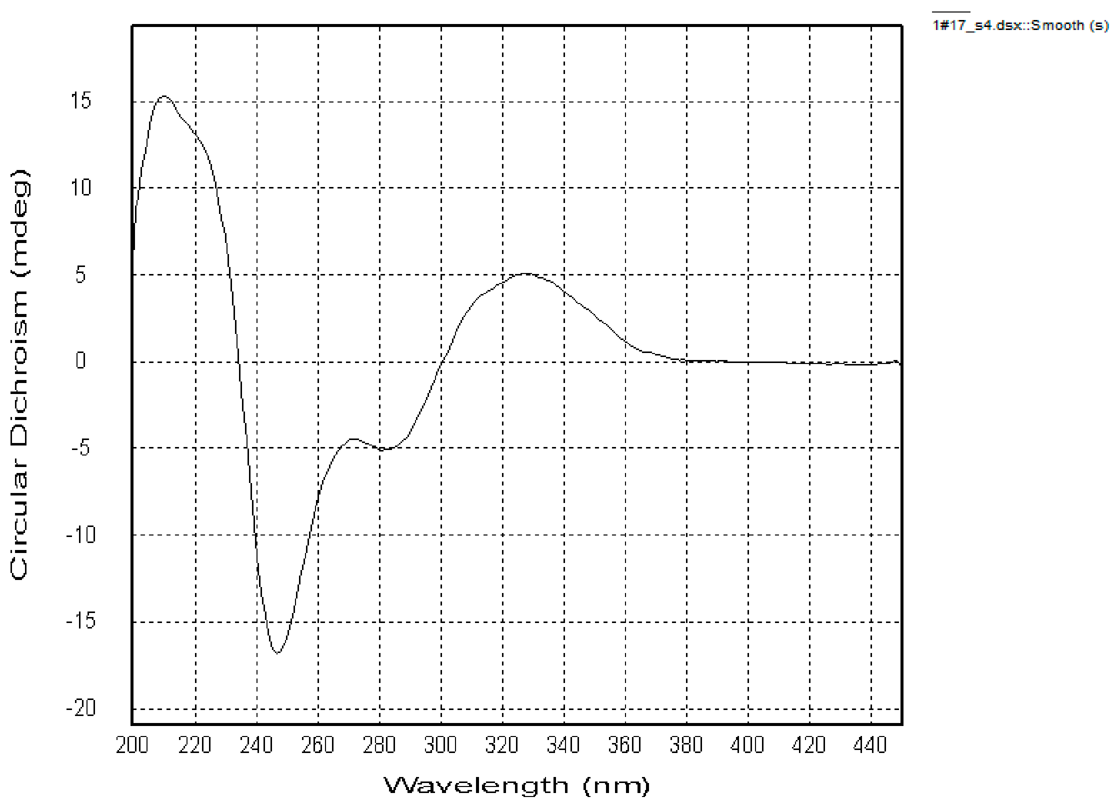

**Figure S9.** $^{1}H$ NMR spectrum (400 MHz, acetone-$d_6$) of compound **1b**

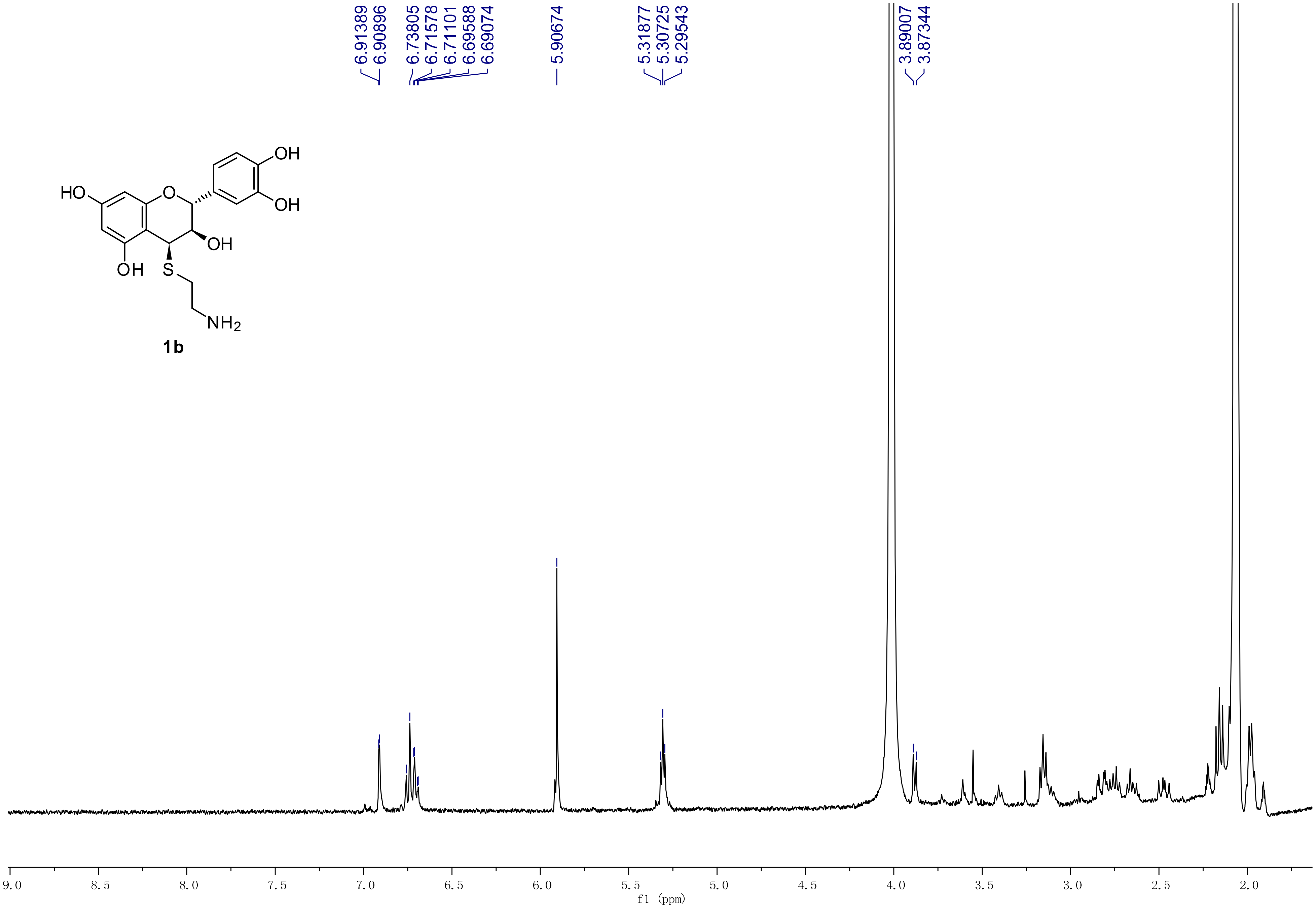
6.91389
6.90896
6.73805
6.71578
6.71101
6.69588
6.69074
5.90674
5.31877
5.30725
5.29543
3.89007
3.87344
HO
O
OH
OH
OH
OH
S
NH2
1b
9.0
8.5
8.0
7.5
7.0
6.5
6.0
5.5
5.0
4.5
4.0
3.5
3.0
2.5
2.0
f1 (ppm)

**Figure S10.** $^{1}H$ NMR spectrum (400 MHz, acetone-$d_6$) of compound **1c**

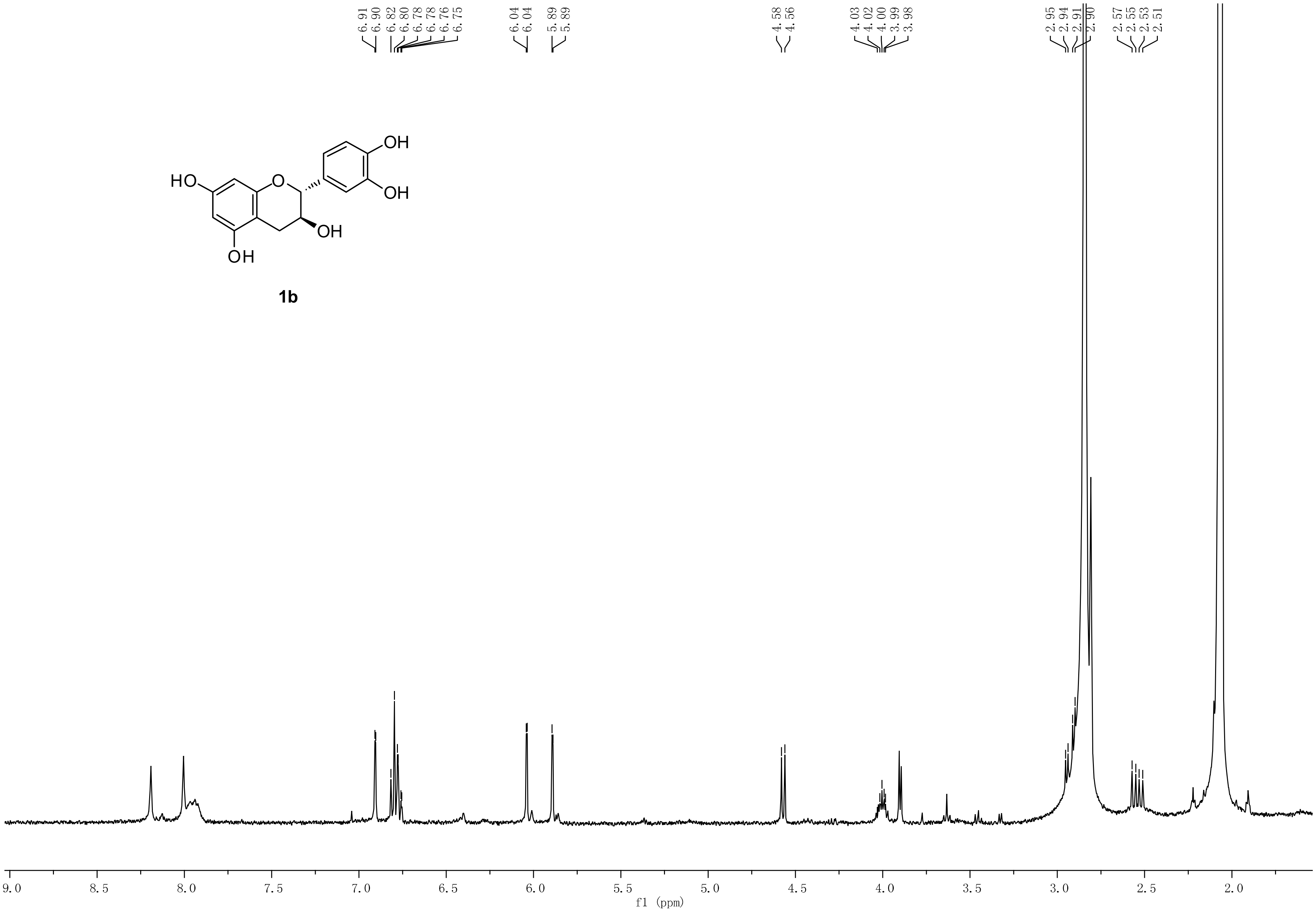
6.91
6.90
6.82
6.80
6.78
6.78
6.76
6.75
6.04
6.04
5.89
5.89
4.58
4.56
4.03
4.02
4.00
3.99
3.98
2.95
2.94
2.91
2.90
2.57
2.55
2.53
2.51
HO
O
OH
OH
OH
OH
1b
9.0 8.5 8.0 7.5 7.0 6.5 6.0 5.5 5.0 4.5 4.0 3.5 3.0 2.5 2.0
f1 (ppm)
